# Epirubicin-loaded marine carrageenan oligosaccharide capped gold nanoparticle system for pH-triggered anticancer drug release

**DOI:** 10.1038/s41598-019-43106-9

**Published:** 2019-05-01

**Authors:** Xiangyan Chen, Wenwei Han, Xia Zhao, Wei Tang, Fahe Wang

**Affiliations:** 1 Key Laboratory of Marine Drugs, Ministry of Education, School of Medicine and Pharmacy, Ocean University of China, Shandong Provincial Key laboratory of Glycoscience and Glycoengineering, Qingdao, 266003 China; 2 Laboratory for Marine Drugs and Bioproducts of Qingdao National Laboratory for Marine Science and Technology, Qingdao, 266237 China; 3 State Key Laboratory of Bioactive Seaweed Substances, Qingdao Brightmoon Seaweed Group Co Ltd, Qingdao, 266400 China

**Keywords:** Target identification, Pharmaceutics

## Abstract

Gold nanoparticles (AuNPs) and the pH stimuli-responsive drug delivery system have been extensively applied in cancer treatment. Carrageenan derived from marine red algae shows a promising application prospect for drug delivery as a nanomaterial for its biodegradability, abundance, and non-toxicity. Carrageenan oligosaccharide (CAO) was used as a biocompatible reductant for green synthesis of CAO-AuNPs, and the obtained CAO-AuNPs were further used as a delivery system for pH-triggered delivery of epirubicin (EPI). The EPI-CAO-AuNPs were demonstrated to be spherical and homogeneous with mean diameter of 141 ± 6 nm by means of electron microscopy and Malvern particle size analyzer. Results showed that the release of EPI from EPI-CAO-AuNPs was significant under acidic condition that simulated cancer environment, while it was negligible under physiological pH *in vitro*. Confocal laser scanning microscope and flow cytometry analysis showed that EPI-CAO-AuNPs were localized in cellular nucleus and induced more apoptosis of HCT-116 and HepG2 cells than free EPI. A new pH-triggered anticancer drug release was achieved by EPI-CAO-AuNPs system for the first time. The developed EPI-CAO-AuNPs nanosystem shows a promising prospect for pH-triggered delivery of antitumor drugs, and our work provides a new idea for targeted drug delivery by using biocompatible marine carbohydrates as nanomaterial.

## Introduction

The targeted drug delivery system has attracted increasing attention in cancer treatment in the last decades. Gold nanoparticles (AuNPs) possess unique physicochemical properties, photothermal effects, biocompatibility and well-controlled size. More importantly, AuNPs can accumulate in target cells through endocytosis and have been used as a targeted carrier for antitumor drug delivery^1–3^. The stimuli-responsive drug delivery system has attracted far-reaching attention for its enhancement in the utilization of drugs by cancer cells, and pH stimuli is a widely used and mainly based on the significant difference of pH between cancer cells and normal cells. Furthermore, the pH stimuli delivery system can be used for intracellular therapeutics by releasing drug in mildly acidic endosomal/lysosomal compartments (pH 4–5)^4^.

**Figure 1:**
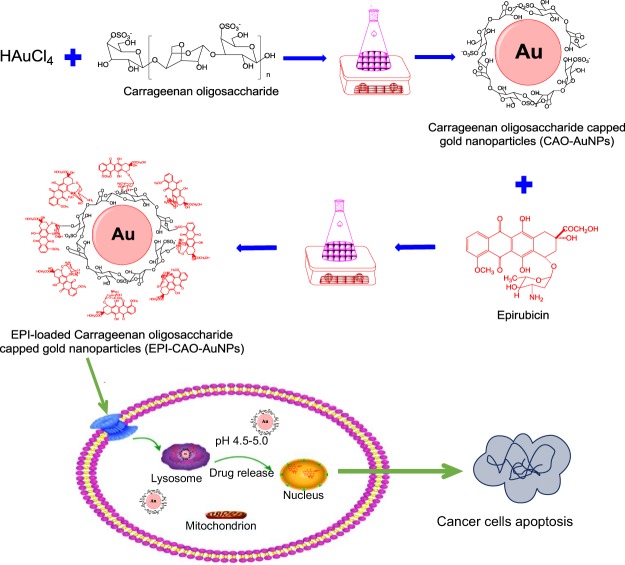
Schematic diagram of EPI-CAO-AuNPs drug delivery system and subsequently pH-triggered drug release under intracellular endo/lysosomal conditions.

Carrageenans are sulfated linear polysaccharides obtained by extraction from marine red algae with a repeating sequence of alternating 3-linked β-D galactopyranose (unit G) and 4-linked α-D-galactopyranose (unit D)^5,6^. In our previous study, we successfully prepared a stable and biocompatible AuNPs by usingcarrageenan oligosaccharide (CAO) derived from carrageenan to act as green reductant as well as stabilizer^7^, and the obtained CAO-AuNPs exhibits a prospective application for drug delivery as a nanomaterial. Among hundreds of developed anthracyclines, epirubicin (EPI) is one of the most widely used in antitumor treatment^8^. However, the poor targeting, cardiotoxicity and allergic reaction of EPI limit its clinical application^9,10^. In present study, EPI was selected as a model drug and loaded in the CAO-AuNPs delivery nano-system to release drug with pH-responsive *in vitro* for the first time. The obtained EPI-CAO-AuNPs nanosystem was characterized and the cellular uptake and anti-cancer activity were also investigated (Fig. 1).

## Methods

### Materials and reagents

Carrageenan extracted from marine red algae was obtained by Qingdao Marine Biomedical Research Institute (Qingdao, China)^7^. Gold (III) chloride trihydrate (HAuCl_4_·3H_2_O) was purchased from Sigma-Aldrich (St.Louis, USA)^7^. Epirubicin Hydrochloride (EPI) was purchased from Zhejiang Hisun Pharmaceutical Co., Ltd (Taizhou, China). HPLC-grade acetonitrile (ACN) and methanol were purchased from Merck KGaA (Germany). Water used in the whole experiments was purified on a Milli-Q system (Millipore, USA). McCOY’S medium (5A) was purchased from HyClone (USA)^7^. Dulbecco’s modified eagle medium (DMEM), L-glutamine, 100 U/mL penicillin and 100 g/mL streptomycin were purchased from Gibco (Grand Island, NY, USA)^7^. Fetal bovine serum (FBS) was purchased from ExCell Bio (Shanghai, China)^7^. All of other chemicals and solvents used were of analytical grade unless otherwise specified^7^. The most of materials and reagents were based on our previous research^7^.

### Preparation of CAO and CAO-AuNPs

The method prepared CAO was described in our previous study^7^. Briefly, CAO was obtained using mild acid hydrolysis of kappa-carrageenan which fractionated with KCl from carrageenan. The kappa-carrageenan (2 g) was hydrolyzed in a solution of HCl (0.1 mol/L, 100 mL) at 60 °C for 1.5 h. The partial hydrolysis was terminated using neutralization with NaOH (1 mol/L), and the hydrolysate was desalted on a Sephadex G10 column (1.6 × 60 cm)^6^. The eluent was collected and concentrated by a rotary evaporator, and then lyophilized to yield a pale yellow solid of CAO. The sulfate content of CAO was determined by a SH-AC-3 anion column (9 µm, 4 mm × 25 mm) on a Shine CIC-100 ion chromatograph (Qingdao, China)^11^. The molecular weight (Mw) of CAO was assayed by a high performance liquid chromatography coupled with refractive index detector (Agilent Technologies, Wilmington, DE, USA) with a column of TSKgel G3000PWXL (TOSOH, Japan). Aqueous Na_2_SO_4_ solution (0.1 mol/L) was used as the mobile phase and the flow rate was 0.5 mL/min. The temperature of column was kept at 35 °C. Dextrans were used as standards to calibrate the column^12^. CAO-AuNPs were prepared as previously published method^7^, as were shown in Fig. 1. Briefly, 0.11 g of CAO was added to 10 mL aqueous solution of HAuCl_4_·3H_2_O (6 × 10^−4^ mol/L), and the solution was kept in magnetic heater stirrer at 50 °C for 3 h to form CAO-AuNPs dispersion by dialysis treatment^7^.

### Preparation of EPI-CAO-AuNPs

The preparation of EPI-CAO-AuNPs was carried as shown in schematic diagram (Fig. 1). Briefly, the prepared CAO-AuNPs (5 mL) was adjusted to pH 9–10 by NaOH solution (0.1 mol/L) and stirred at 40 °C for 20 min. Then a solution containing EPI (3 mL, 0.8 mg/mL) was added dropwise into CAO-AuNPs, and the reaction was incubated at 40 °C for 24 h. Finally, orange red dispersion occurred which indicated that EPI-CAO-AuNPs successfully synthesized. The EPI-CAO-AuNPs dispersion was dialyzed by means of dialysis membrane (MWCO, 14 kDa) to remove free EPI and CAO-AuNPs, and then stored at 4 °C and kept out of the light until required.

### Drug entrapment efficiency and loading efficiency

In order to analyze the loading efficiency of EPI, the unloaded drug concentration of EPI-CAO-AuNPs was determined by means of HPLC (Agilent Technologies 1260, USA) at wavelength of 233 nm. A Zorbax-Extend-C_18_ column (4.6 mm × 150 mm, 5 μm, Agilent Technologies, USA) was used for determination of EPI with acetonitrile and water as a mobile phase. The volume ratio of acetonitrile/water was 30/70 with 0.1% trifluoroacetic acid (TFA), the column temperature was remained at 25 °C, and the flow rate was 1.0 mL/min. Methanol (10 mL) was added into 500 μL of EPI-CAO-AuNPs dispersion to make it disintegrate for releasing the loaded EPI drug. Then the mixture was oscillated for 1 min, sonicated for 5 min, and centrifuged at 10,000 rpm for 10 min. The process was repeated as above steps one time. The supernatant solution was collected and measured to calculate the residual content of EPI. The entrapment efficiency (EE) and drug loading efficiency (LE) percentages were calculated according to the following equations: EE (%, w/w) = (Mass of drug in nanoparticles/Mass of initial added drug) × 100; LE (%, w/w) = (Mass of drug in nanoparticles/Mass of nanoparticles) × 100.

### Characterization of EPI-CAO-AuNPs

Some characterization methods of EPI-CAO-AuNPs were referred to our previous study in characterization of CAO-AuNPs^7^. The morphology of EPI-CAO-AuNPs was characterized by a JEM-2100EX transmission electron microscopy (TEM), a high resolution transmission electron microscopy (HRTEM) (Jeol, Tokyo, Japan), and a JSM-6700F scanning electron microscopy (SEM) (Jeol, Tokyo, Japan). The mean diameter, polydispersity index (PDI) and zeta potentials (ZP) of EPI-CAO-AuNPs nanoparticles were performed using a Nano-ZS90 Malvern particle size analyzer (Malvern Instruments Ltd, United Kingdom) at a scattering angle of 90° and at room temperature. The Fourier transform infra-red (FT-IR) spectra of EPI-CAO-AuNPs and CAO-AuNPs powders which prepared by lyophylization were performed by means of a Nexus 470 spectrophotometer (Nicolet, San Diego, CA, USA) with KBr pellets operated at a resolution of 2 cm^−1^ of wavelength in the range 4000–400 cm^−1^. The X-ray diffraction (XRD) measurement was obtained by preparing a thin film of powdered EPI-CAO-AuNPs on a D-MAX 2500/PC XRD diffractometer (Rigaku, Tokyo, Japan)^3,13^. Differential scanning thermogram (DSC) analysis of EPI-CAO-AuNPs powder was carried out under nitrogen atmosphere with a heating rate at 10 °C/min from 50 to 900 °C using a thermo gravimetric analyzer (TA Instruments, SDTQ 600, Schaumburg, IL, USA). The changes in UV-Vis spectrum of CAO-AuNPs and EPI-CAO-AuNPs dispersions were recorded up to 6 months and 3 months, respectively, using a UV–vis spectroscopy 2800 (Shimadzu, Kyoto, Japan) with quartz cell at a resolution of 2 nm^7^.

### Cell culture and viability assay

The human hepatoma cells (HepG2), human colon cancer cells (HCT-116) and human umbilical vein endothelial cells (HUVEC) were purchased from the Shanghai Cell Bank of Chinese Academy of Sciences. All cells were grown in DMEM, and supplemented with 10% heat-inactivated FBS, 2 mM L-glutamine, 100 U/ml penicillin and 100 g/ml streptomycin in a humidified 5% CO_2_ cell culture incubator at 37 °C^7^. The cells used in all experiments were in the logarithmic phase of growth. The HepG2 cells and HUVEC cells were seeded into 96-well culture plates, and incubated for 24 h to make cell adhere^7^. After completed adherence of cells, the culture medium was treated with different concentrations of EPI, CAO-AuNPs and EPI-CAO-AuNPs (0.0156, 0.0312, 0.0625, 0.125, 0.250, 0.500 μmol/L) in separate wells for 72 h incubation to perform cytotoxic analysis using sulforhodamine B (SRB) colorimetric assay. After SRB staining in each well, a solution of Tris was added into the cultures (150 μL/well) and the absorbance was measured to determine cell viability at 540 nm in a multi-well ELISA plate reader (Molecular Devices, USA).

### Cell uptake assay

The HCT-116 cells were cultured at a density of 1.2 × 10^5^ per well into 20 mm glass bottom culture dishes, and allowed to attach the glass substrate for 3 h after 1.8 mL supplemented growth medium was added. The cells were incubated at 37 °C with 5% CO_2_ for 24 h, and then free EPI and EPI-CAO-AuNPs (equal to 0.25 μmol/L) were added into the corresponding cell wells and incubated for 4 h in dark. After designated incubating, the culture medium was removed and washed with PBS (pH 7.4) three times. Then, 4% formaldehyde was used to fix the cells for 30 min and the cells were washed twice with PBS (pH 7.4). Subsequently, 4,6-diamidino−2 -phenylindole dihydrochloride (DAPI, Sigma-Aldrich, USA) was added to the plates to stain the nuclei in dark, and the uptake of fixed cells was observed at 480 ± 20 nm by a Confocal Laser Scanning Microscope (CLSM, Zeiss LSM 510 Meta, Germany).

### Flow cytometry analysis

The HCT-116 cells and HepG2 cells were plated in 6-well plates (2 × 10^5^ cells/well) at 37 °C for 24 h. Then they were incubated in dark for 24 h and 48 h, with or without free EPI and EPI-CAO-AuNPs, for cell apoptosis analysis. While the cells were incubated in dark for 24 h for cell cycle analysis. After that, the cells were collected with 0.25% trypsin, centrifuged at 1200 rpm for 5 min, and rinsed with PBS. Then, the cells were fixed with ice-cold (70%) ethanol at −20 °C to stand overnight. After incubation, the cells were centrifuged at 1200 rpm for 5 min, and the pellet was washed with PBS. Then the cells were resuspended and stained with 5 μL Annexin V-FITC and 5 μL Propidium Iodide (PI)^14^. After being gently oscillated, the cells were kept in room temperature for 15 min in dark. After being gently oscillated, the cell apoptosis was measured by means of flow cytometry (FCM, Beckman Coulter, Miami, FL, USA). The data were analyzed by Summit 5.2 software (Beckman Coulter, Inc. USA). As for cell cycle analysis, the cells were stained with PI and ribonuclease A, and incubated at 4 °C for 30 min in dark^4^. The cell cycle stage was analyzed from cell population by means of FCM.

### pH-triggered drug release *in vitro*

The pH dependent behavior of EPI-CAO-AuNPs was investigated under physiological condition (pH 7.4) and simulated cancerous condition (pH 5.0) *in vitro* for 72 h, respectively. Briefly, the EPI-CAO-AuNPs (2 mL) was loaded into a dialysis membrane (MWCO, 3500 Da) and placed in 30 mL of phosphate buffered saline (PBS, pH 7.4 & 5.0) with continuous stirring at 37 °C. 1 mL of solution from each buffer was harvested with subsequent replacement of equal volume fresh buffer at the designated time interval. The concentration of EPI release was analyzed by means of HPLC at 233 nm, and the experiments were performed in triplicates for each sample.

### Statistical analysis

All experiments data were displayed as the mean ± standard deviations (SD), and the significance was calculated by means of GraphPad Prism 5.0 software (GraphPad Software, San Diego, CA, USA). Comparison between groups was performed by using One-Way ANOVA analysis followed by tukey’s test. The P values < 0.05 was considered to be significant, and P values < 0.01 was supposed to be highly significant.

## Results and Discussion

### Synthesis and Characterization of EPI-CAO-AuNPs

**Figure 2:**
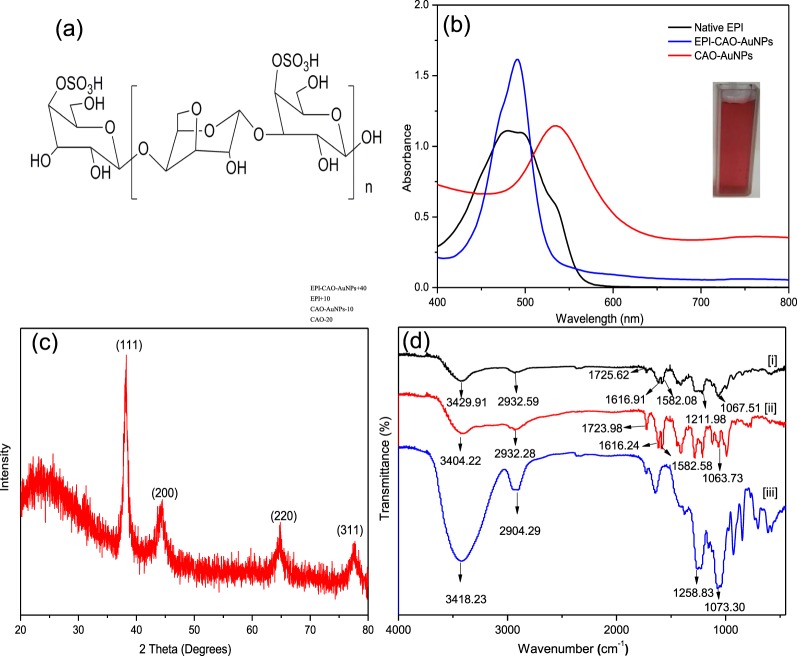
Chemical structure of CAO and Characterization of EPI-CAO-AuNPs: (**a**) Structure of CAO; (**b**) UV-Vis spectra analysis of native EPI solution, re-dispersed pellet of EPI-CAO-AuNPs and supernatant of EPI-CAO-AuNPs, and the inset photograph showed orange red color of EPI-CAO-AuNPs dispersion; (**c**) XRD pattern of EPI-CAO-AuNPs; (**d**) FT-IR spectra of EPI-CAO-AuNPs (i), EPI (ii), and CAO-AuNPs (iii).

CAO (kappa-CAO) was obtained from mild acid hydrolysis of kappa-carrageenan verified as the repeating disaccharide in Fig. 2a ^6^, and the used method is easy and effective. The Mw and sulfate content of CAO were 1200 Da and 22.3%, respectively. A green synthesis method was employed to obtain CAO-AuNPs by using CAO as a reducing and stabilizing agent^7^. The overall synthetic process did not use any chemical toxic reagents, and the obtained CAO-AuNPs remained stable for 6 months without aggregation (Additional Information: Fig. S1). The CAO-AuNPs system was adjusted to pH 9–10 and epirubicin hydrochloride (EPI^.^HCl) was added drop by drop into the system to produce a high-load non-ionized EPI nanosystem^15^. Furthermore, the negatively charged CAO-AuNPs could electrostatically interact with positively charged EPI under alkaline conditions, and the H-bonds were also generated between protonated N atom of EPI and -OH of CAO-AuNPs^16,17^, which all were beneficial to form stable EPI-CAO-AuNPs system. The EE and LE of EPI-CAO-AuNPs measured by a HPLC method were 94.3% and 12.5%, respectively. It was observed that EPI-CAO-AuNPs exhibited a strong peak at 490 nm (Fig. 2b), which was different from the absorbance of CAO-AuNPs at 530 nm, indicating that EPI drug was successfully conjugated onto the surface of CAO-AuNPs. The UV-Vis spectrum of EPI-CAO-AuNPs had no shift over several weeks when stored at 4 °C in dark (Additional Information: Fig. S2), and the EPI-CAO-AuNPs solution showed no aggregation, indicating that EPI-CAO-AuNPs formed was stable. As shown in Fig. 2c, The intense diffraction peaks from the XRD pattern of EPI-CAO-AuNPs powder were observed at 2θ degree of 38.22°, 44.48°, 64.84°, and 77.63°, which corresponded to the (111), (200), (220), and (311) reflection of the crystalline metallic gold, respectively, indicated that obtained EPI-CAO-AuNPs were crystalline^3,18^. The functional groups and interactions of EPI-CAO-AuNPs were confirmed by FT-IR analysis (Fig. 2d). FT-IR spectrum of EPI (ii) and EPI-CAO-AuNPs (i) showed that the absorption band of NH_2_ stretching vibration of EPI was shifted from 3404.22 to 3429.91 cm^−1^, the peak of C=O stretching of EPI was shifted from 1723.98 to 1725.62 cm^−1^, the band of -C=N stretching of EPI aromatic ring was shifted from 1616.24 to 1616.91 cm^−1^, and the peak of C-O-C stretching of EPI was shifted from 1063.73 to 1067.51 cm^−1^ ^19^. The shifts in characteristic absorption peaks revealed that changed functional groups (-NH_2,_ -C=O, -C=N, -C-O-C) of EPI had a certain relationship with the synthesis of EPI-CAO-AuNPs. Furthermore, the -SO_3_^−^ stretching peak of CAO-AuNPs (iii) was also shifted from 1258.83 cm^−1^ to 1211.98 cm^−1^ ^20^, indicating that the -SO_3_^−^ groups of CAO-AuNPs were associated with the ammonium groups of EPI by electrostatic interaction to form EPI-CAO-AuNPs. In addition, the EPI-CAO-AuNPs showed principal FT-IR peaks of both CAO-AuNPs and EPI, indicating that the EPI was loaded onto CAO-AuNPs nanoparticles by means of non-covalent binding^21^.

**Figure 3:**
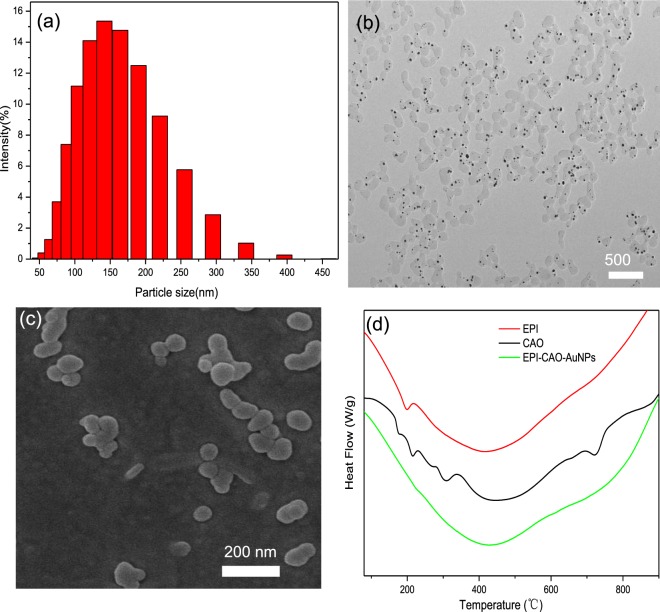
Characterization of EPI-CAO-AuNPs: (**a**) particle size distribution graph; (**b**) TEM image of EPI-CAO-AuNPs; (**c**) SEM image of EPI-CAO-AuNPs; (d) DSC curves of EPI, CAO and EPI-CAO-AuNPs.

The size and morphology of AuNPs play important roles in celluar uptake and treatment efficacy^22,23^. The particle size in the range of 100–200 nm can avoid phagocytosis of immune system by receptor-mediated endocytosis and prolong their circulation time in vivo to increase the efficacy^24^. As shown in Fig. 3, the particle average size of EPI-CAO-AuNPs obtained by Nano-ZS90 Marvin particle size analyzer was 141 ± 6 nm (Fig. 3a) with 0.15 ± 0.01 of PDI, indicating that the size distribution of EPI-CAO-AuNPs was homogeneous and easy to be absorbed by cells through endocytosis. The TEM image of EPI-CAO-AuNPs (Fig. 3b) demonstrated that the NPs were mono-dispersed. It was showed that EPI part was light-colored transparent around dark gold core, indicating that EPI was successfully loaded onto the surface of CAO-AuNPs. The morphology of EPI-CAO-AuNPs as observed by SEM (Fig. 3c) showed a spherical and ellipsoidal shape from a stereoscopic perspective. DSC is a useful method to evaluate the phase transition and thermal stability of AuNPs. As illustrated in Fig. 3d, the pure EPI and native CAO showed endothermic transition at 415 °C and 450 °C, respectively. While the EPI-CAO-AuNPs displayed endothermic peak at 424 °C, indicating that EPI was loaded on the surface of EPI-CAO-AuNPs instead of being present as molecular or amorphous dispersion^21^. Furthermore, the thermal stability of EPI-CAO-AuNPs was decreased compared with that of CAO, which might be due to that EPI changed the structure and properties of CAO^19,25^.

### Cell cytotoxicity of EPI-CAO-AuNPs on normal and cancerous cells

**Figure 4:**
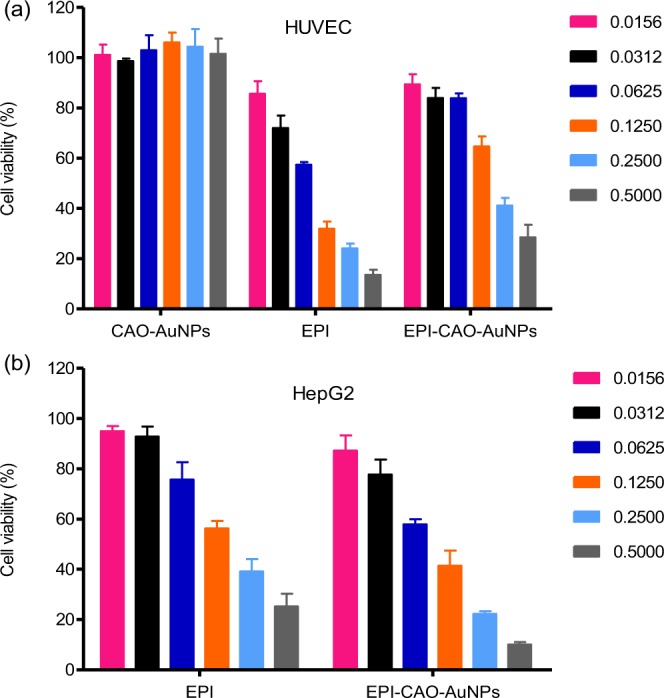
Cell viability assay of CAO-AuNPs, EPI and EPI-CAO-AuNPs on noncancerous and cancerous cells at different concentrations (0.0156–0.500 μmol/L) for 72 h by the SRB: (**a**) HUVEC cell and (**b**) HepG2 cell in a dose-dependent manner (mean ± SD, n = 3).

The cytotoxicity of CAO-AuNPs, EPI and EPI-CAO-AuNPs was evaluated using normal (HUVEC: Fig. 4a) and cancerous cells (HepG2: Fig. 4b) by the SRB assay, respectively. It was observed that the normal cell viability of CAO-AuNPs group was all more than 95% in different concentrations (Fig. 4a), which revealed that green obtained CAO-AuNPs had good biocompatibility. The obtained EPI-CAO-AuNPs nanosystem exhibited lower cytotoxic activity onto normal cells than that of free EPI, indicated that EPI-CAO-AuNPs reduced the cytotoxicity of free EPI to normal cells. As illustrated in Fig. 4b, both EPI and EPI-CAO-AuNPs exhibited a dose-dependent decrease in cell viability against HepG2 cells over a dose range of 0.0156–0.5000 μmol/L, and the inhibition of EPI-CAO-AuNPs was more significant than that of EPI at the same dose. The EPI-CAO-AuNPs onto HepG2 cells yielded an IC_50_ value of 0.087 ± 0.036 μmol/L significantly lower than that of free EPI, which yielded a value of 0.173 ± 0.043 μmol/L after 72 h exposure. This result indicated that EPI-CAO-AuNPs showed stronger cytotoxic activity against tumour cells than free EPI, which may be beneficial for the specificity of AuNPs to enhance cellular uptake and increase drug bioavailability^2,4,26^. In addition, CAO with a certain degree of anticancer activity might play a synergistic effect in combination with EPI^27,28^. Hence, the EPI-CAO-AuNPs displayed potential values for cancer treatment.

### The localization of EPI-CAO-AuNPs in cells by confocal imaging

**Figure 5:**
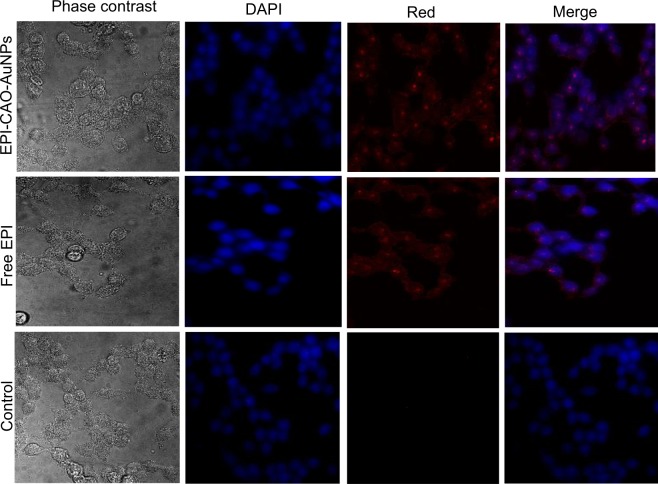
CLSM images of HCT116 cells incubated with EPI-CAO-AuNPs and free EPI at 37 °C for 4 h and DAPI at room temperature for 10 min.

To investigate the localization of EPI-CAO-AuNPs in the cells, the ability of intracellular drug delivery of EPI-CAO-AuNPs was evaluated in HCT-116 cells by means of CLSM (Fig. 5). Only blue fluorescence produced by DAPI staining for nuclei was observed in the control group of HCT-116 cells, indicating that the absence of EPI which produced a red-colored fluorescence. However, the red fluorescence was detected in the cytoplasm and nucleus after the cells were incubated with free EPI or EPI-CAO-AuNPs for 4 h, respectively. More importantly, the EPI-CAO-AuNPs group showed stronger red fluorescence than that of free EPI, indicating that EPI-CAO-AuNPs were more easily absorbed by cells via endocytosis compared with free EPI^29^. Furthermore, the red fluorescence overlapped with the blue fluorescence in EPI-CAO-AuNPs group indicated that EPI which was released from EPI-CAO-AuNPs was entered into nucleus.

### Cell cycle assay on cancer cells induced by EPI-CAO-AuNPs

**Figure 6:**
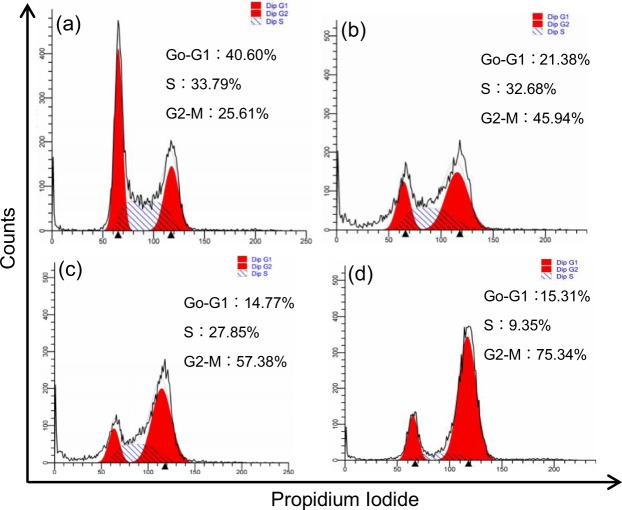
Effects of EPI-CAO-AuNPs on cell cycle analysis of HCT-116 cells cultured for 24 h using PI staining: (**a**) untreated control, (**b**) 0.25 μM of free EPI, (**c**) 0.25 μM of free EPI-CAO-AuNPs, (**d**) 0.5 μM of free EPI-CAO-AuNPs.

Abnormal cell cycle of tumor cells, especially G2/M phase, is particularly important to maintain malignant proliferation. The regulation of EPI-CAO-AuNPs on the cell cycle of HCT-116 and HepG2 cells was investigated on a flow cytometry by means of PI staining (as shown in Fig. 6 and Additional Information: Fig. S3, respectively). The accumulation of HCT-116 cells in the G2/M region caused by free EPI, EPI-CAO-AuNPs at low dose and high dose was 45.94%, 57.38% and 75.34%, respectively, which were all higher than 25.61% in the control group (Fig. 6). These results proved that EPI-CAO-AuNPs induced cell cycle arrest significantly at the G2/M phase in both HCT-116 and HepG2 cells. Furthermore, the EPI-CAO-AuNPs induced cell apoptosis in a dose-dependence and more significant than free EPI (Fig. 6 and Additional Information: Fig. S3), which was consistent with results of SRB assay.

### Cell apoptosis assay on cancer cells induced by EPI-CAO-AuNPs

**Figure 7:**
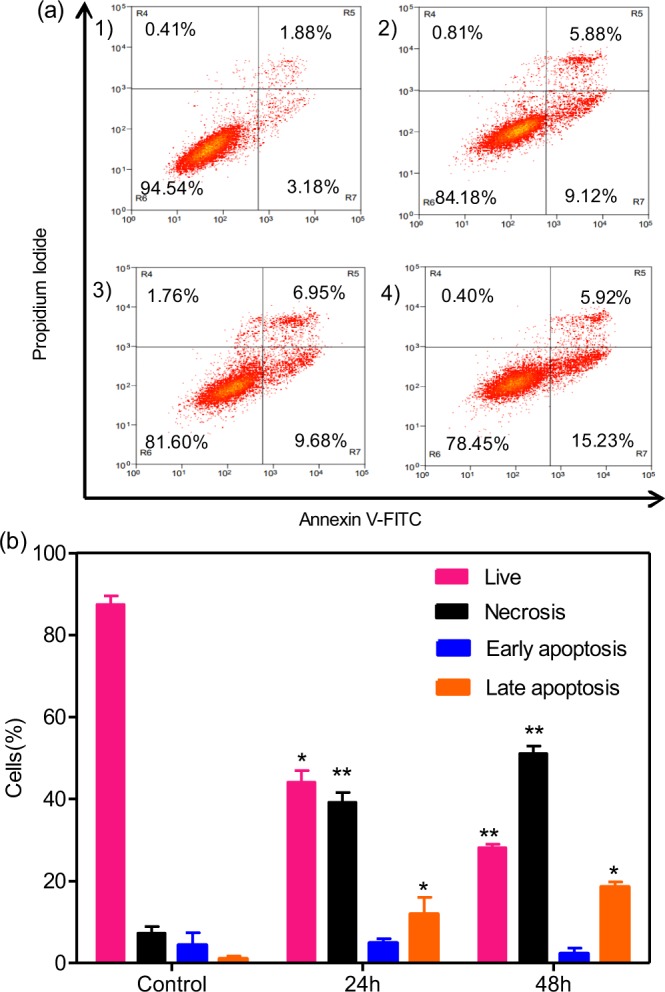
Flow cytometry analysis results using Annexin V-FITC and PI staining: (**a**), Cell apoptosis in HCT-116 cells incubated with (1) untreated control; (2) 0.25 μM of free EPI for 48 h; (3) 0.25 μM of free EPI-CAO-AuNPs for 48 h; (4) 0.5 μM of free EPI-CAO-AuNPs for 48 h; (**b**), Bar graph of (3) illustrating the mean percentages of apoptosis and necrosis (*significant p < 0.05, **highly significant p < 0.01).

Annexin V-FITC/PI double staining was applied to evaluate the apoptosis of EPI-CAO-AuNPs against HCT-116 cells & HepG2 cells using flow cytometry (as shown in Fig. 7 and Additional Information: Fig. S4, respectively). All cells were treated with a dose above or below IC_50_ value of free EPI and EPI-CAO-AuNPs for 48 h. It was observed that EPI-CAO-AuNPs induced more apoptosis and necrosis on the cells compared with free EPI, which was in a concentration-dependent manner and was consistent with results of SRB assay (Fig. 7a and Additional Information: Fig. S4). When HCT-116 cells were incubated with a close IC_50_ dose of EPI-CAO-AuNPs for 24 h & 48 h, the mean percentage of Annexin V-FITC/PI staining negative cells (live cells) significantly decreased from 94.54% to 81.60% or 78.45%, respectively (Fig. 7b). More importantly, the percentages of live and necrotic cells in HCT-116 cells incubated with a close IC_50_ concentration of EPI-CAO-AuNPs for 48 h were 81.60% and 1.76%, respectively. However, the percentages of live and necrotic cells in HepG2 cells were 28.7% and 52.4%, respectively. This results might be due to the fact that EPI-CAO-AuNPs system contains CAO, which is mainly composed of galactose, can specifically recognize the galactose or N-acetylgalactosamine residue of asialoglycoprotein receptor (ASGP-R) which only exists on the surface of hepatocytes or hepatocellular carcinoma cell membrane^30^. Hence, the EPI-CAO-AuNPs produced higher cytotoxicity to HepG2 cells with ASGP-R than HCT-116 cells without ASGP-R.

### Release characteristics of EPI-CAO-AuNPs

**Figure 8:**
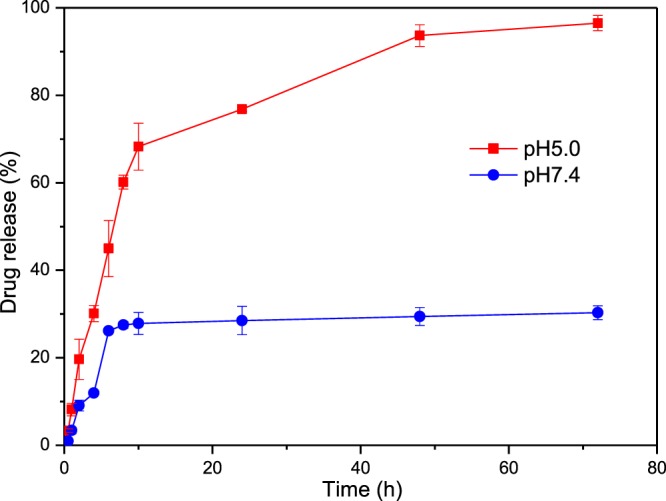
pH dependent percentage release of EPI from EPI-CAO-AuNPs nanosystem under different conditions.

We chose EPI as a cancer model drug and investigated the drug release of EPI-CAO-AuNPs at different pH conditions (as shown in Fig. 8). The EPI-CAO-AuNPs nanosystem was incubated in a neutral PBS solution (pH 7.4) which was to simulate the physiological environment, and in an acidic PBS solution (pH 5.0) which was to simulate the endosomal/lysosomal compartment and cancer tissue environment *in vitro*^31,32^. As observed from Fig. 8, EPI were released 96% from EPI-CAO-AuNPs after 72 h at pH 5.0, however, only 30% of EPI were released after 72 h at pH 7.4. Thus, the release of EPI under acidic condition (pH 5.0, 37 °C) was three times faster than under the physiologic condition (pH 7.4, 37 °C). These data indicated that EPI-CAO-AuNPs nanosystem was more easily released under acidic conditions than that in neutral pH, which is beneficial to reduce the toxicity of EPI on normal tissue^33^. The EPI drug release characteristic might be related with the CAO in EPI-CAO-AuNPs nanosystem, which is mainly composed of galactose and can specifically recognize the galactose or N-acetylgalactosamine residue of asialoglycoprotein receptor (ASGP-R) on the surface of cancer cells^2,30^. In addition, the dissociation degree of sulfates of CAO in EPI-CAO-AuNPs would decrease in an acidic condition, which would weaken the H-bond interaction between EPI and CAO to facilitate EPI release from EPI-CAO-AuNPs. These results were consistent with that from the localization of EPI-CAO-AuNPs by CLSM, and further confirmed that the lower pH in cancer cells triggered the EPI release from EPI-CAO-AuNPs. Thus, EPI-CAO-AuNPs might initially enter the cytoplasm, and accumulated in lysosome (pH 4.5–5.0) to achieve EPI drug release, then the drug subsequently entered into the nucleus^31,34^.

## Conclusions

A new pH-triggered anticancer drug release was achieved by EPI-CAO-AuNPs system for the first time, which prepared by a green and simple method using CAO extracted from marine carrageenan as a biocompatible nanomaterial. The EPI-CAO-AuNPs were proved to be spherical as well as homogeneous with mean diameter of 141 ± 6 nm, and the drug loading efficiency and entrapment efficiency was 12.5% and 94.3%, respectively. The release rate of EPI from EPI-CAO-AuNPs was faster in simulated cancerous cells environment with low pH value than that in neutral pH. The EPI-CAO-AuNPs were initially entered the cytoplasm and maybe accumulate in the lysosome (pH 4.5–5.0) to achieve EPI release, and induced cell apoptosis at the G2/M phase in both HCT-116 and HepG2 cells. Furthermore, EPI-CAO-AuNPs exhibited significantly stronger cytotoxicity and induced more apoptosis and necrosis on cancer cells than that on normal cells, compared with free EPI. Hence, the EPI-CAO-AuNPs nanosystem shows a promising prospect for targeted drug delivery, and our work provides a new idea for targeted drug delivery by using biocompatible marine carbohydrates as nanomaterial.

## Supplementary information

Supplementary Information

## References

[1] Hu Panpan, Li Gang, Zhao Xia, Zhao Fengli, Li Liangjie, Zhou Houcheng (2018). Transcriptome profiling by RNA-Seq reveals differentially expressed genes related to fruit development and ripening characteristics in strawberries (Fragaria × ananassa). PeerJ.

[2] Wang SH, Lee CW, Chiou A, Wei PK (2010). Size-dependent endocytosis of gold nanoparticles studied by three-dimensional mapping of plasmonic scattering images. J Nanobiotechnology.

[3] Manivasagan P (2016). Doxorubicin-loaded fucoidan capped gold nanoparticles for drug delivery and photoacoustic imaging. Int J Biol Macromol.

[4] Ghorbani M, Hamishehkar H (2017). Decoration of gold nanoparticles with thiolated pH-responsive polymeric (PEG-b-p(2-dimethylamio ethyl methacrylate-co-itaconic acid) shell: A novel platform for targeting of anticancer agent. Mater Sci & Engineer C Mater for Biol Applica.

[5] Vanessaleiria C, Danielfábio K, Dílsonbrazdajr S, Ivone C (2009). Carrageenans: Biological properties, chemical modifications and structural analysis–A review. Carbohydr Polym.

[6] Yu GL (2006). Sequence determination of sulfated carrageenan-derived oligosaccharides by high-sensitivity negative-ion electrospray tandem mass spectrometry. Analytical Chem.

[7] Chen Xiangyan, Zhao Xia, Gao Yanyun, Yin Jiaqi, Bai Mingyue, Wang Fahe (2018). Green Synthesis of Gold Nanoparticles Using Carrageenan Oligosaccharide and Their In Vitro Antitumor Activity. Marine Drugs.

[8] Zagotto G, Gatto B, Moro S, Sissi C, Palumbo M (2001). Anthracyclines: recent developments in their separation and quantitation. J Chromatogra B.

[9] Devi PR, Kumar CS, Selvamani P, Subramanian N, Ruckmani K (2015). Synthesis and characterization of Arabic gum capped gold nanoparticles for tumor-targeted drug delivery. Mater Lett.

[10] Lo YL, Ho CT, Tsai FL (2008). Inhibit multidrug resistance and induce apoptosis by using glycocholic acid and epirubicin. Eur J Pharm Sci.

[11] Xue YT (2016). Study on quality control of sulfated polysaccharide drug, propylene glycol alginate sodium sulfate (PSS). Carbohydr Polym.

[12] Zhao X (2007). Preparation of low-molecular-weight polyguluronate sulfate and its anticoagulant and anti-inflammatory activities. Carbohydr Polym.

[13] Nascente PAP (2006). Structure, morphology, and composition of nanometric Pd films deposited by dc magnetron sputtering on Cu, Ag, and Au foils. Mater Sci & Engineer A.

[14] Xia F (2018). Cytokine induced killer cells-assisted delivery of chlorin e6 mediated self-assembled gold nanoclusters to tumors for imaging and immuno-photodynamic therapy. Biomaterials.

[15] Zhang G, Hubalewska M, Ignatova Z (2009). Transient ribosomal attenuation coordinates protein synthesis and co-translational folding. Nature Struc & Molec Biolo.

[16] Mohanty RK, Thennarasu S, Mandal AB (2014). Resveratrol stabilized gold nanoparticles enable surface loading of doxorubicin and anticancer activity. Colloid & Surf B Biointerf.

[17] Tomoaia G (2015). Effects of doxorubicin mediated by gold nanoparticles and resveratrol in two human cervical tumor cell lines. Colloid & Surf B Biointerf.

[18] Gutiérrezwing C, Esparza R, Vargashernández C, García MEF, Joséyacamán M (2012). Microwave-assisted synthesis of gold nanoparticles self-assembled into self-supported superstructures. Nanoscale.

[19] Laksee S, Puthong S, Teerawatananond T, Palaga T, Muangsin N (2017). Highly efficient and facile fabrication of monodispersed Au nanoparticles using pullulan and their application as anticancer drug carriers. Carbohydr Polym.

[20] Ahmed KB, Kalla D, Uppuluri KB, Anbazhagan V (2014). Green synthesis of silver and gold nanoparticles employing levan, a biopolymer from Acetobacter xylinum NCIM 2526, as a reducing agent and capping agent. Carbohydr Polym.

[21] Pooja D, Panyaram S, Kulhari H, Rachamalla SS, Sistla R (2014). Xanthan gum stabilized gold nanoparticles: characterization, biocompatibility, stability and cytotoxicity. Carbohydr Polym.

[22] Banerjee A, Qi J, Gogoi R, Wong J, Mitragotri S (2016). Role of Nanoparticle Size, Shape and Surface Chemistry in Oral Drug Delivery. J Control Release.

[23] Zhang XD (2012). Size-dependent radiosensitization of PEG-coated gold nanoparticles for cancer radiation therapy. Biomaterials.

[24] Win KY, Feng SS (2005). Effects of particle size and surface coating on cellular uptake of polymeric nanoparticles for oral delivery of anticancer drugs. Biomaterials.

[25] Trovatti E (2012). Sustainable nanocomposite films based on bacterial cellulose and pullulan. Cellulose.

[26] Patra CR, Bhattacharya R, Mukhopadhyay D, Mukherjee P (2008). Application of Gold Nanoparticles for Targeted Therapy in Cancer. J Biomedical Nanotech.

[27] Hayashi K, Nakamura M, Ishimura K (2013). Near-Infrared Fluorescent Silica-Coated Gold Nanoparticle Clusters for X‐Ray Computed Tomography/Optical Dual Modal Imaging of the Lymphatic System. Adv Healthcare Mater.

[28] Yuan H, Song J, Li X, Li N, Dai J (2006). Immunomodulation and antitumor activity of kappa-carrageenan oligosaccharides. Cancer Lett.

[29] Patra S (2015). Green synthesis, characterization of gold and silver nanoparticles and their potential application for cancer therapeutics. Mater Sci & Engineer C Mater for Biolog Applicat.

[30] Yen HJ (2018). Positively charged gold nanoparticles capped with folate quaternary chitosan: Synthesis, cytotoxicity, and uptake by cancer cells. Carbohydr Polym.

[31] Cheng R, Meng F, Deng C, Klok HA, Zhong Z (2013). Dual and multi-stimuli responsive polymeric nanoparticles for programmed site-specific drug delivery. Biomaterials.

[32] Yang Y (2017). Gold nanoparticle-gated mesoporous silica as redox-triggered drug delivery for chemo-photothermal synergistic therapy. J Colloid & Interf Sci.

[33] Aryal S, Grailer JJ, Pilla S, Steeber DA, Gong S (2009). Doxorubicin conjugated gold nanoparticles as water-soluble and pH-responsive anticancer drug nanocarriers. J Mater Chem.

[34] Khutale GV, Casey A (2017). Synthesis and characterization of a multifunctional gold-doxorubicin nanoparticle system for pH triggered intracellular anticancer drug release. Eur J Pharma & Biopharma.

