# Supplementary material for: Epirubicin-loaded marine carrageenan oligosaccharide capped gold nanoparticle system for pH-triggered anticancer drug release
Source: Sci Rep. 2019 May 1;9:6754. doi: 10.1038/s41598-019-43106-9 (PMC6494808; doi:10.1038/s41598-019-43106-9)
Supplement: Supplementary file 1 — Supplementary Information [file 41598_2019_43106_MOESM1_ESM.pdf]

Epirubicin-loaded marine carrageenan oligosaccharide capped gold nanoparticle system for pH-triggered anticancer drug release

Xiangyan Chen[1,2], Wenwei Han[1,2], Xia Zhao[1,2*], Wei Tang[1] & Fahe Wang[3]

[1]Key Laboratory of Marine Drugs, Ministry of Education, School of Medicine and Pharmacy, Ocean University of China, Shandong Provincial Key laboratory of Glycoscience and Glycoengineering, Qingdao 266003, China

[2]Laboratory for Marine Drugs and Bioproducts of Qingdao National Laboratory for Marine Science and Technology, Qingdao 266237, China

[3]State Key Laboratory of Bioactive Seaweed Substances, Qingdao Brightmoon Seaweed Group Co Ltd, Qingdao 266400, China

* **Corresponding Author**: Xia Zhao

School of Medicine and Pharmacy, Ocean University of China,

Qingdao 266003, P. R. China

Tel. /fax: +86-532-8203-1560,

E-mail: 1184748799@qq.com; zhaoxia@ouc.edu.cn(X. Zhao)

Supplementary Figures

Supplementary Figures S1

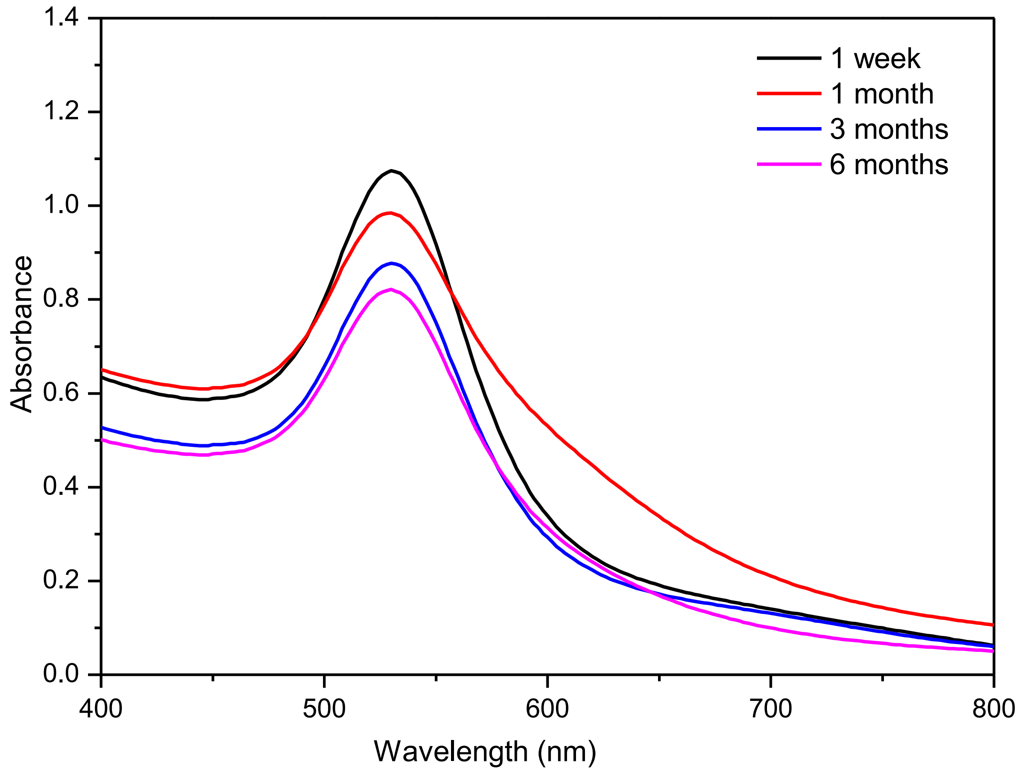

Fig. S1. UV-Vis absorption spectra of CAO-AuNPs in six months stability study.

Supplementary Figures S2

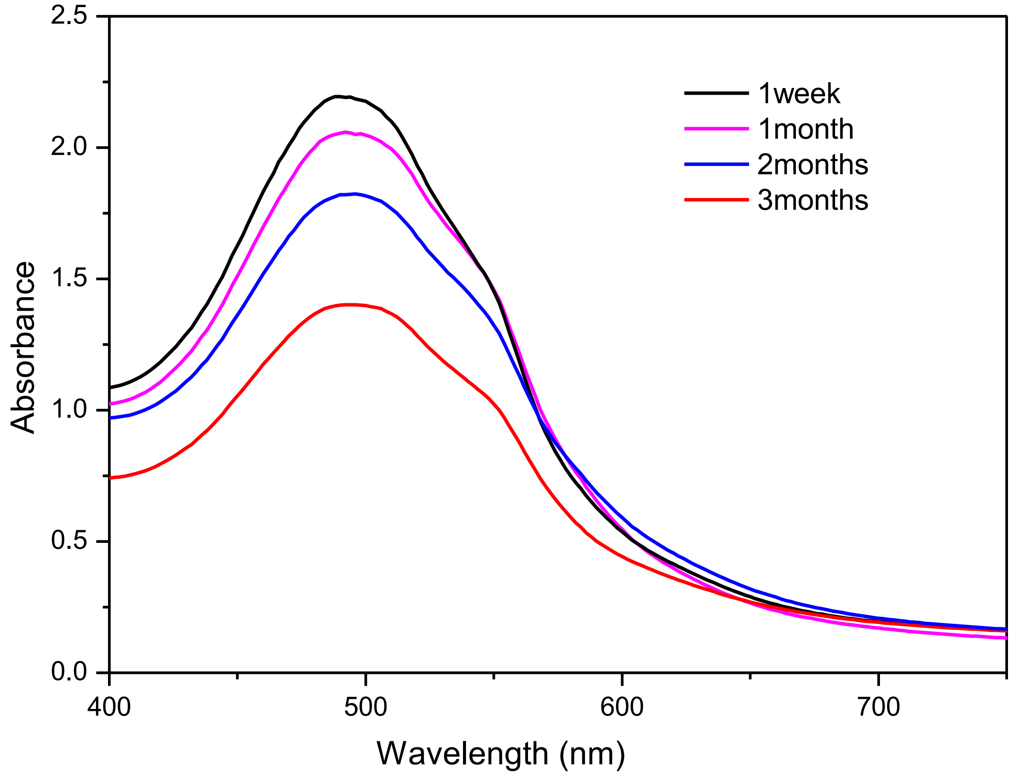

Fig. S2. UV-Vis absorption spectra of EPI-CAO-AuNPs in three months stability study.

Supplementary Figures S3

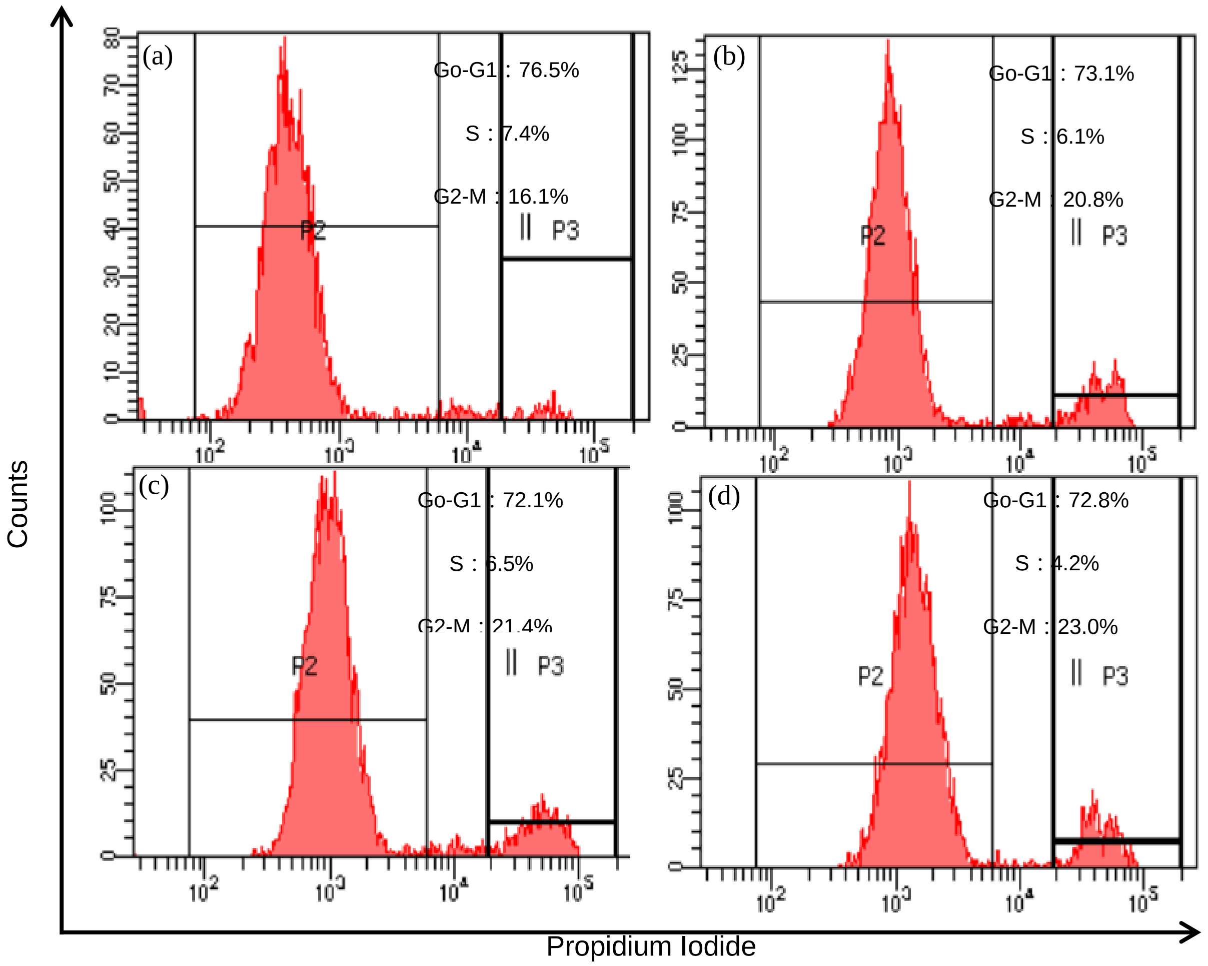

Fig. S3. Effects of EPI-CAO-AuNPs on cell cycle analysis of HepG2 cells cultured for 24 h using PI staining: (a) untreated control, (b), 0.1 μM of free EPI, (c), 0.1 μM of free EPI-CAO-AuNPs, (d), 0.2 μM of free EPI-CAO-AuNPs.

Supplementary Figures S4

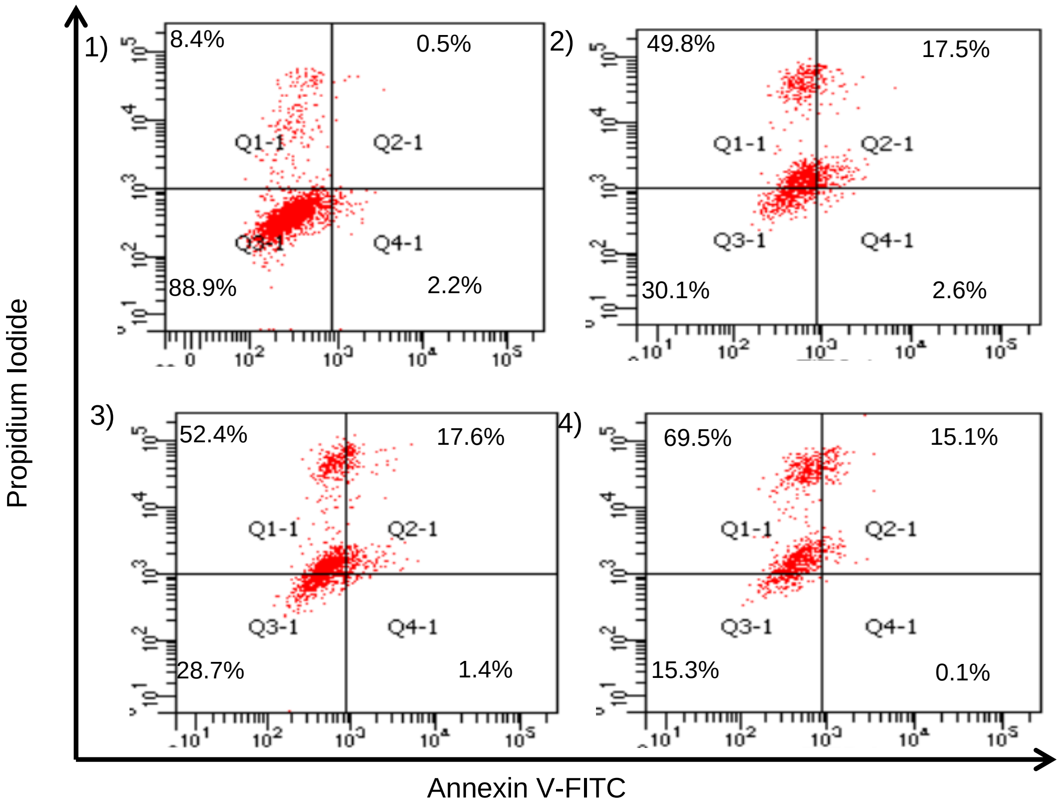

Fig. S4. Flow cytometry analysis of cell apoptosis in HepG2 cells incubated with, 1), untreated control; 2), 0.1 μM of free EPI; 3), 0.1 μM of EPI-CAO-AuNPs; 4), 0.2 μM of EPI-CAO-AuNPs, for 48 h using Annexin V-FITC and PI staining.
